# Germline copy number variants in *RUNX1*: An updated case report and a decade-old red herring

**DOI:** 10.1038/s44276-024-00117-y

**Published:** 2025-03-27

**Authors:** Natalie T. Deuitch, Amra Kajdic, Erica Bresciani, Marshall S. Horwitz, Hamish S. Scott, Katie Craft, Shawn Chong, David J. Young, Lucy A. Godley, Paul P. Liu

**Affiliations:** 1 Oncogenesis and Development Section, Translational and Functional Genomics Branch, National Human Genome Research Institute, NIH, Bethesda, MD USA; 2 Laboratory Medicine & Pathology, University of Washington School of Medicine, Seattle, WA USA; 3 Centre for Cancer Biology, SA Pathology and University of South Australia, Adelaide, SA Australia; 4 Translational Stem Cell Biology Branch, National Heart Lung and Blood Institute, NIH, Bethesda, MD USA; 5 Robert H. Lurie Comprehensive Cancer Center, Division of Hematology/Oncology, Northwestern University, Evanston, IL USA

## Abstract

Pathogenic/likely pathogenic (P/LP) germline variants in *RUNX1* cause familial platelet disorder with associated myeloid malignancies (FPDMM), also known as *RUNX1-*Familial Platelet Disorder (RUNX1-FPD, or FPD), a condition characterized by qualitative and quantitative platelet defects and predisposition to hematopoietic malignancies. Here, we present follow up to a case of a woman with acute myeloid leukemia and lifelong thrombocytopenia which had previously been attributed to presumptive pathogenic (P) *GATA2* missense variants. However, re-evaluation with updated molecular technology sensitive for detection of copy number variants (CNVs) led to the identification of a P deletion of exons 5-6 in *RUNX1*, which had been undetected when examined at first presentation. This case highlights the importance of comprehensive molecular evaluation and careful variant interpretation, especially regarding CNVs.

## Introduction

Familial platelet disorder with associated myeloid malignancies (FPDMM), also known as RUNX1-Familial Platelet Disorder (RUNX1-FPD, or FPD), is an autosomal dominant condition caused by deleterious *RUNX1* variants and characterized by quantitative and qualitative platelet defects as well as a 35–45% lifetime risk of hematopoietic malignancies [1, 2]. *RUNX1* encodes a master hematopoietic transcription factor, which plays important roles in cell cycle control and embryogenesis.

*RUNX1* is one of a growing number of genes associated with predisposition to hematopoietic malignancies, including *ETV6, ANKRD25*, and *DDX41*. Early and accurate identification of these conditions can have important clinical implications for patients and their family members, especially in selection of donors for hematopoietic cell transplant (HCT) [3]. Diagnosis can be difficult as pathogenic/likely pathogenic (P/LP) germline variants in *ETV6* and *ANKRD26* phenocopy many aspects of FPDMM, most notably constitutional thrombocytopenia. However, the hematopoietic malignancies in these conditions differ. People with FPD typically develop myeloid malignancies, but lymphoid malignancies have been reported. In contrast, P/LP *ETV6* variants more frequently cause B-cell ALL, and P/LP ANKRD26 variants have only been associated with myeloid malignancies [4]

Like *RUNX1*, *GATA2* also encodes a master hematopoietic transcription factor. GATA2 deficiency leads to a broad spectrum of phenotypes, including immunodeficiency, lymphedema, pulmonary involvement, congenital deafness, and a strong predisposition to early onset hematopoietic malignancies. Monosomy 7 is a common cytogenic alteration in GATA2 deficiency and is noted in up to 60% of individuals with AML or MDS [5, 6].

Here, we describe the re-evaluation of a pedigree, in which the original proband’s condition was previously reported as GATA2 deficiency [7, 8]. However, use of comprehensive cancer risk testing in a family member provided an alternate diagnosis of RUNX1-FPDMM.

## Case

In 2011, a 50-year-old woman was diagnosed with acute myeloid leukemia (AML) with monosomy 7, and was reported to have GATA2 deficiency after molecular profiling on blood identified two germline *GATA2* variants (p.Thr358Lys and p.Leu359Val) in the second zinc finger which were found to reside *in cis*. She had been noted to have a longstanding history of thrombocytopenia, which is atypical for GATA2 deficiency. She died due to complications of allogeneic HCT using a matched unrelated donor (MUD).

Her case was published in two separate reports as an example of GATA2 deficiency, with novel *in cis* variants [7, 8]. One of the variants, p.Leu359Val, had been previously been reported in recurrent somatic chronic myeloid leukemia (CML) progression, but had not previously been seen in the germline. In vitro studies showed that together, the two variants impacted protein function and were presumed to be pathogenic [7, 8]. Familial segregation of the *GATA2* variants was not performed as the family had declined testing.

Twelve years later, the proband’s brother was diagnosed with myelodysplastic syndrome (MDS) at 58 years of age. Next generation sequencing (NGS) of the MDS revealed somatic *DNMT3A*, *TET2* and *ZRSR2* mutations, and the patient was referred for germline cancer risk testing given his personal and family history of MDS/AML. Germline testing of DNA from cultured skin fibroblasts reveled a deletion of exons 5-6 of the *RUNX1* gene, classified as P by ACMG Myeloid Malignancy Variant Curation Expert Panel (MM-VCEP) criteria [9]. Of note, the *RUNX1* deletion was not detected by somatic NGS tumor profiling. However, the GATA2 variants originally identified in his sister were not detected.  The patient underwent a MUD HCT and is doing well almost a year post-transplant.

**Fig. 1:**
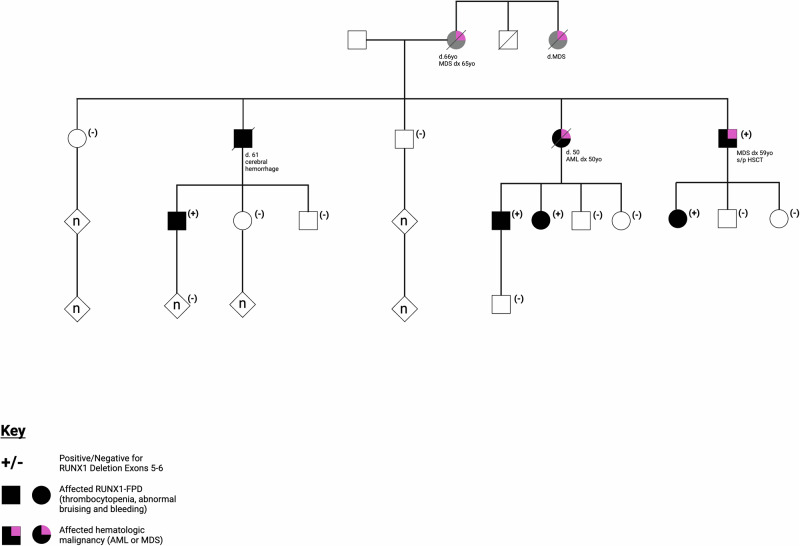
Family history with segregation of the *RUNX1* deletion. Squares represent males and circles represent females; non-reported sex is indicated by diamonds. Fully shaded symbols represent individuals diagnosed with RUNX1-FPDMM, all of whom have symptoms of thrombocytopenia and abnormal bruising. Individuals with grey shading are suspected to have had RUNX1-FPDMM, however were unavailable for testing. Pink shading represents individuals with hematologic malignancy (AML or MDS). Labeling with (+) and (−) indicates that individuals were confirmed to be positive or negative for the *RUNX1* deletion of exons 5-6 via NGS. Some pedigree details have been altered for patient confidentiality. Created with BioRender.com.

Cascade testing for the family was performed, and the *RUNX1* variant was found to segregate with individuals in the family who exhibited thrombocytopenia, abnormal bleeding, and bruising (Fig. 1). Notably, a brother, and obligate carrier of the variant, died of a sudden cerebral hemorrhage, a rare but known complication of FPDMM. All affected members of this family are now being followed on the NIH RUNX1 Natural History Study and/or by local hematologists. Monitoring may vary by individual, but this typically consists of annual bone marrow biopsies and complete blood counts every 3–6 months.

Through familial segregation, the original proband was identified as an obligate carrier for the *RUNX1* deletion. Looking back, the studies done on peripheral blood when she presented did not evaluate for copy number variants (CNVs), which were thought to be rare at the time. Consequently, the *RUNX1* deletion was missed, and her life-long thrombocytopenia and AML were inappropriately attributed to the germline *GATA2* variants.

## Discussion

**Fig. 2:**
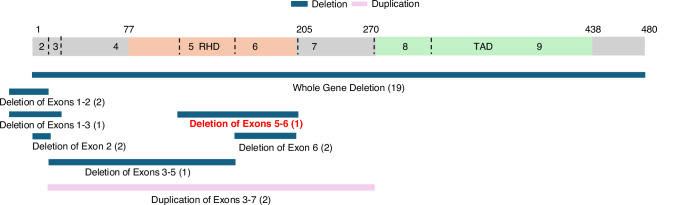
*RUNX1* copy number variants (CNVs) reported in the Natural History Study to date. Blue bars indicate deletions and the pink bar represent a duplication. Numbers in brackets designate the number of families reported with a given deletion, though exact genomic bounds may vary. This family’s deletion (exons 5-6) is highlighted with red font. This figure represents the CNVs reported in Cunningham et al. [1], as well as additional CNVs that have been identified since. 30 of the 86 total LP/P variants that have been identified to date are CNVs.

We now recognize that CNVs are common in *RUNX1*-FPDMM [1, 10] and account for more than 25% of variants reported in the NIH RUNX1 Natural History Study (ClinicalTrials.gov ID: NCT03854318) (Fig. 2). Partial CNVs can be found throughout the *RUNX1* gene. Partial duplications have been reported and appear to lead to loss of functional protein [10]. In some cases, the whole *RUNX1* gene may be deleted, sometimes as a part of a 21q contiguous gene deletion syndrome. CNVs are commonly missed on clinical testing, especially when the initial test is a somatic mutation panel and may result in delayed or even missed diagnoses. When this family first presented, CNVs were thought to be rare. However, incorporation of microarrays and advances in sequencing technologies and bioinformatic pipelines into comprehensive clinical cancer risk testing have resulted in the increased recognition of these variants. It is essential that CNVs, as well as intronic variants, are always evaluated in cases with a clinical suspicion of RUNX1-FPDMM.

**Fig. 3:**
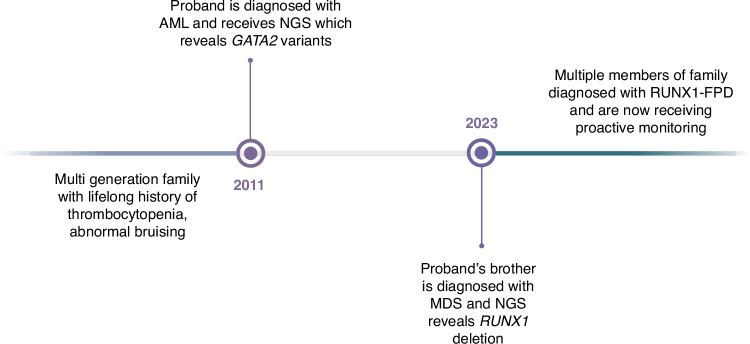
Case timeline. Created with BioRender.com.

This case also highlights the importance of ongoing and thorough evaluation – and reevaluation – of families with a suspected clinical disease. Monosomy 7 is a hallmark feature of GATA2 deficiency but has also been reported in cases of RUNX1-FPDMM. Although thrombocytopenia has been noted in individuals with GATA2 deficiency, it is typically within the context of other cytopenias, most commonly neutropenia [5, 6]. The role of the cis *GATA2* variants in the original proband’s malignancy has yet to be determined. Because the original DNA sample was obtained from blood, it is not possible to determine if the *GATA2* variants were germline or somatic. The combination of a germline *RUNX1* variant in the setting of either germline or acquired *GATA2* mutation may explain the severity of the proband’s disease. However, ultimately, it was *RUNX1*, not *GATA2* variants, that segregated with disease, demonstrating the importance of cascade testing to substantiate risk associations (Fig. 3).

This case shows how re-evaluation of a family with new sequencing technologies led to a more accurate diagnosis and has now enabled members of the family to access proactive monitoring for their disease.

## Data Availability

No datasets were generated or analysed during the current study.
